# Supplementary material for: Clinical characteristics and risk factors for coronavirus disease 2019 (COVID-19) among patients under investigation in Thailand
Source: PLoS One. 2020 Sep 15;15(9):e0239250. doi: 10.1371/journal.pone.0239250 (PMC7491739; doi:10.1371/journal.pone.0239250)
Supplement: Attachment — Submitted filename: PO SK COVID PUI Response to reveiwer.docx [file pone.0239250.s002.docx]

Dear the editor and reviewers:

Thank you, the editors and referees, for your review of our manuscript submission and insightful comments. Please see the revised manuscript and response below.

Sincerely yours,

Sasisopin Kiertiburanakul, MD, MHS

Reviewer Comments:

**Comments to the Author**

1. Is the manuscript technically sound, and do the data support the conclusions?

The manuscript must describe a technically sound piece of scientific research with data that supports the conclusions. Experiments must have been conducted rigorously, with appropriate controls, replication, and sample sizes. The conclusions must be drawn appropriately based on the data presented.

Reviewer #1: Yes

Reviewer #2: Yes

2. Has the statistical analysis been performed appropriately and rigorously?

Reviewer #1: Yes

Reviewer #2: Yes

3. Have the authors made all data underlying the findings in their manuscript fully available?

The [PLOS Data policy](http://www.plosone.org/static/policies.action#sharing) requires authors to make all data underlying the findings described in their manuscript fully available without restriction, with rare exception (please refer to the Data Availability Statement in the manuscript PDF file). The data should be provided as part of the manuscript or its supporting information, or deposited to a public repository. For example, in addition to summary statistics, the data points behind means, medians and variance measures should be available. If there are restrictions on publicly sharing data—e.g. participant privacy or use of data from a third party—those must be specified.

Reviewer #1: Yes

Reviewer #2: Yes

4. Is the manuscript presented in an intelligible fashion and written in standard English?

PLOS ONE does not copyedit accepted manuscripts, so the language in submitted articles must be clear, correct, and unambiguous. Any typographical or grammatical errors should be corrected at revision, so please note any specific errors here.

Reviewer #1: Yes

Reviewer #2: Yes

5. Review Comments to the Author

Please use the space provided to explain your answers to the questions above. You may also include additional comments for the author, including concerns about dual publication, research ethics, or publication ethics. (Please upload your review as an attachment if it exceeds 20,000 characters)

Reviewer #1: The article addresses the timely and critical information on the clinical characteristics and risk factors for COVID-19, among the PUIs. The findings of the study add so much of valuable information to medical literature. The article has been written well and data have been analysed appropriately.

However, following are the minor comments to be addressed by the authors:

1. The retrospective review has been done in PUIs covering a period of only 2 weeks. Though the authors have mentioned that this was when there were highest rates of cases reported. It would have added more strength to the analysis had more patients been included.

**Answer:** Our study was covered for only 2-week period due to an epidemiological characteristic of COVID-19 in Thailand is relatively brief. Therefore, we tentatively selected this specific period when the majority of PUIs attended healthcare facilities for an investigation when the information regarding some specific risk factors was completely collectable and deem interpretable. We noted this limitation in the discussion section for the authors. See page 16, line 334-338.

2. In both the PUIs and the COVID-19 positive population, there is a majority of female population. This is unlike the reports from other countries such as China and India. Can the authors throw more light on this gender difference observed in this study population as against other geographical locations?

**Answer:** We added the following explanation in the discussion section see page 13-14, line 273-279. An unexpected caveat is although the male gender predominance was observed among several cohorts regarding vulnerability to COVID-19, our result instead revealed female gender is more frequently diagnosed. A reason to explain this disparity has been proposed but not entirely clear, however, an outcome seemed indifferent. Furthermore, a greater proportion of female PUIs in our cohort likely from a coincidence or possibly more attention in their health conditions was more prominent among the female population [19, 20].

19) Gebhard C, Regitz-Zagrosek V, Neuhauser HK, Morgan R, Klein SL. Impact of sex and gender on COVID-19 outcomes in Europe. *Biol Sex Differ*. 2020;11(1):29. Published 2020 May 25. doi:10.1186/s13293-020-00304-9

20) Jin JM, Bai P, He W, et al. Gender Differences in Patients With COVID-19: Focus on Severity and Mortality. *Front Public Health*. 2020;8:152.

3. Patients not being on medical coverage is being found to be an important risk factor owing to the lower socioeconomic status and crowded living conditions. That brings up the concern on whether the close contacts of those patients with no medical coverage were traced and tested?

**Answer:** A national policy is also implemented to monitor those who were closely contacted with an index patient (COVID-19 patient) and complimentarily investigated for COVID-19 should new symptoms occur. We added this information in the discussion section. See page 13, line 267-269.

4. In line numbers 105 to 108, there is mention of including cases that did not match the case definition. But there is no mention of the numbers of this category that were included.

**Answer:** There were 347 (85.7%) and 58 (14.3%) patients who were fulfilled the criteria and designated as a PUI, respectively. We added this information in the result section. Page 9, line 171-172.

5. Also, there is no breakup of the numbers of the PUIs being classified as severe and non-severe cases.

**Answer:** Twenty-six (6.4%) severe PUIs and 379 (93.6%) non-severe PUIs were classified as the aforementioned criteria. We added this information in the result section. Page 9, line 172-173.

6. Line 135 &136 mentions collection of endotracheal aspirates from patients who were intubated. Wondering how many and why were they being intubated even before specimen collection status?

**Answer:** Five (1.2%) patients underwent endotracheally intubation on arrival due to acute respiratory failure and therefore endotracheal aspirates were collected accordingly. We added the following sentences “Among 400 (98.8%) patients underwent nasopharyngeal and throat swabs and 5 (1.2%) patients provided endotracheal aspirates for SARS-CoV-2 PCR” We added this information in the result section. Page 9, line 175-176.

7. What is the fate of the majority of the PUIs tested negative for COVID-19? What could have contributed to the fever, respiratory symptoms among them? Were they tested for other respiratory conditions?

**Answer:** Among 352 (86.9%) PUIs who did not have a positive result for COVID-19, 40 (11.4%) patients underwent further investigations. There were seven patients in the non-COVID-19 group diagnosed with infections with non-COVID pathogens: influenza virus (n=2), *Pneumocystis jirovecii* (n=1), *Haemophilus influenzae* (n=2), *Klebsiella pneumoniae* (n=1), and *Staphylococcus aureus* (n=1). We added this information in the result section. Page 11, line 220-224.

**Reviewer #2:** This manuscript titled 'Clinical Characteristics and Risk Factors for Coronavirus Disease 2019 (COVID-19) among Patients under Investigation in Thailand' is very well written and has useful information in the current situation. Its interesting to see that few of the patients had life threatening, other co-morbidity along with the COVID-19.

I understand that lot of background information is collected from the patients in the hospital. I am not sure if some of it is relevant for the scientific discussion part of this paper. For ex. the religion of the patients. I have hard time figuring out why is it mentioned in the paper if its not discussed anywhere in detail. Same is the case for mentioning percentage of male patient when there is not significant correlation. Thanks.

**Answer:** Since there is no correlation of religions and COVID-19, therefore we decided to remove this information. We added the following explanation regarding male sex in the discussion section see page 13-14, line 273-279. An unexpected caveat is although the male gender predominance was observed among several cohorts regarding vulnerability to COVID-19, our result instead revealed female gender is more frequently diagnosed. A reason to explain this disparity has been proposed but not entirely clear, however, an outcome seemed indifferent. Furthermore, a greater proportion of female PUIs in our cohort likely from a coincidence or possibly more attention in their health conditions was more prominent among the female population [19, 20].

19) Gebhard C, Regitz-Zagrosek V, Neuhauser HK, Morgan R, Klein SL. Impact of sex and gender on COVID-19 outcomes in Europe. *Biol Sex Differ*. 2020;11(1):29. Published 2020 May 25. doi:10.1186/s13293-020-00304-9

20) Jin JM, Bai P, He W, et al. Gender Differences in Patients With COVID-19: Focus on Severity and Mortality. *Front Public Health*. 2020;8:152.
